# Diversification of the Type VI Secretion System in Agrobacteria

**DOI:** 10.1128/mBio.01927-21

**Published:** 2021-09-14

**Authors:** Chih-Feng Wu, Alexandra J. Weisberg, Edward W. Davis, Lin Chou, Surtaz Khan, Erh-Min Lai, Chih-Horng Kuo, Jeff H. Chang

**Affiliations:** a Department of Botany and Plant Pathology, Oregon State University, Corvallis, Oregon, USA; b Molecular and Cellular Biology Program, Oregon State University, Corvallis, Oregon, USA; c Institute of Plant and Microbial Biology, Academia Sinica, Taipei, Taiwan; Brigham and Women's Hospital/Harvard Medical School

**Keywords:** evolution, competition, type VI secretion system, pathogen, *Agrobacterium*

## Abstract

The type VI secretion system (T6SS) is used by many Gram-negative bacteria to deploy toxic effectors for interbacterial competition. This system provides a competitive advantage *in planta* to agrobacteria, a diverse group with phytopathogenic members capable of genetically transforming plants. To inform on the ecology and evolution of agrobacteria, we revealed processes that diversify their effector gene collections. From genome sequences of diverse strains, we identified T6SS loci, functionally validated associated effector genes for toxicity, and predicted genes homologous to those that encode proteins known to interact with effectors. The gene loci were analyzed in a phylogenetic framework, and results show that strains of some species-level groups have different patterns of T6SS expression and are enriched in specific sets of T6SS loci. Findings also demonstrate that the modularity of T6SS loci and their associated genes engenders dynamicity, promoting reshuffling of entire loci, fragments therein, and domains to swap toxic effector genes across species. However, diversification is constrained by the need to maintain specific combinations of gene subtypes, congruent with observations that certain genes function together to regulate T6SS loading and activation. Data are consistent with a scenario where species can acquire unique T6SS loci that are then reshuffled across the genus in a restricted manner to generate new combinations of effector genes.

## INTRODUCTION

The type VI secretion system (T6SS) is a membrane-associated apparatus used by Gram-negative bacteria often to compete and cooperate with other bacteria for access to space and nutrients (1). T6SSs are assembled from three subcomplexes and resemble an inverted phage taillike structure. The contractile taillike subcomplex engages with other cells and consists of an outer sheath and an inner Hcp (hemolysin-coregulated protein) tube, capped by VgrG (“spike”) along with a PAAR (proline-alanine-alanine-arginine; “spike-sharpening”) protein, all of which can be associated with effector proteins. Upon contraction of the outer sheath, the Hcp tube is propelled out, and components of the contractile taillike subcomplex are released out.

Against other bacteria, T6SS-associated effectors typically target general features of cells and therefore expose attackers to risks of self-intoxication. To protect themselves, attacker cells carry immunity genes cognate to each effector gene (EI pair). Toxic effectors and immunity proteins have important impacts on the ecology and evolution of bacteria. Deployment of toxic effectors can give aggressors a competitive advantage over others or the ability to segregate communities into cooperative patches (2–4). Processes that result in immunity, or alter the composition of immunity genes, impact social dynamics of bacterial communities (5–7).

Horizontal gene transfer (HGT) is hypothesized to diversify EI pairs. Members of some species of bacteria have numerous modular effector genes that belong to a single class, and effector genes can be swapped or are susceptible to recombination that exchanges C-terminal-encoded toxin domains (8, 9). In other bacterial species, EI pairs are located within *vgrG* loci that include VgrG-, PAAR-, and Tap-encoding genes (10–13). Tap proteins are adapters, with either a DUF4123, DUF2169, or DUF1715 domain, that load cognate effectors onto specific VgrG-PAAR variants (14–16). Hence, the organization of *vgrG* loci links genes that encode interacting proteins. Yet recombination is hypothesized to occur at any site in these loci and generate new partner combinations, mosaics, and arrays of orphan immunity genes (17). It is unknown how restriction of *vgrG* loci to certain gene combinations is balanced against their diversification.

The agrobacteria-rhizobia complex (ARC) is a genus-level group that consists of a polyphyletic group of plant-pathogenic agrobacteria intermixed with nitrogen-fixing rhizobia that also includes *Shinella* (18, 19). Agrobacteria are divided into three main lineages predicted to have emerged independently and at different times in the history of the genus. One lineage consists predominantly of biovar 1 (BV1), which is subdivided into many genomospecies that are mostly commensurate to species-level groups and is sister to narrow-host-range species of agrobacteria (19–22). The second lineage consists of two species-level groups called biovar 2 (BV2) and biovar 2-like (BV2-like). The third lineage is biovar 3 (BV3), and it circumscribes multiple undefined species-level groups. In the first and third major lineages, members have two chromosomes and diverse plasmids, while in the second, members have only a single chromosome and diverse plasmids (23–25).

The T6SS provides some BV1 strains a competitive advantage in culture and *in planta* (13, 26). In strains analyzed to date, T6SS genes cluster into two kinds of T6SS loci (13, 27, 28). The large locus is predicted to consist of two operons. The conserved *imp* operon encodes regulatory proteins and components that complete or belong in each of the three T6SS subcomplexes (29–31). The *hcp* operon encodes proteins that constitute the contractile taillike subcomplex and includes a *tai-tae*, *tap*, and another EI pair. The *tai-tae* pair encode an immunity protein and a peptidoglycan amidase effector, respectively, that is more conserved than the *tap* and EI pair, which are polymorphic across strains and organized downstream of a cognate *vgrG* gene (13, 22, 32). The second T6SS locus is the accessory *vgrG* locus that includes *vgrG*, *tap*, and an EI pair. For example, in reference strain C58 (BV1; G8), *vgrG1* is downstream of *hcp* and associated with *tap1* (DUF4123), while *vgrG2* is in an accessory locus and associated with *tap2* (DUF2169) (29). Across agrobacterial strains, accessory *vgrG* loci can vary in composition (13, 22, 33).

We uncovered the genetic and phenotypic diversity of T6SSs to inform on processes that promote and constrain EI diversification in a genus-level group of bacteria. We reanalyzed whole-genome sequences from strains across the ARC, focusing largely on a subset of strains that was originally analyzed for the purposes of developing an evolutionary framework for agrobacteria (19). Findings from combined computational and experimental studies were consistent in showing that HGT and recombination lead to the acquisition of novel loci and generation of new gene combinations. Findings also showed that diversification of T6SS loci is offset by the need to retain specific gene and domain combinations within loci, and recombination tends to concentrate at specific hot spots or occur between loci similar in gene composition.

## RESULTS

### The large T6SS locus is dynamic.

**FIG 1:**
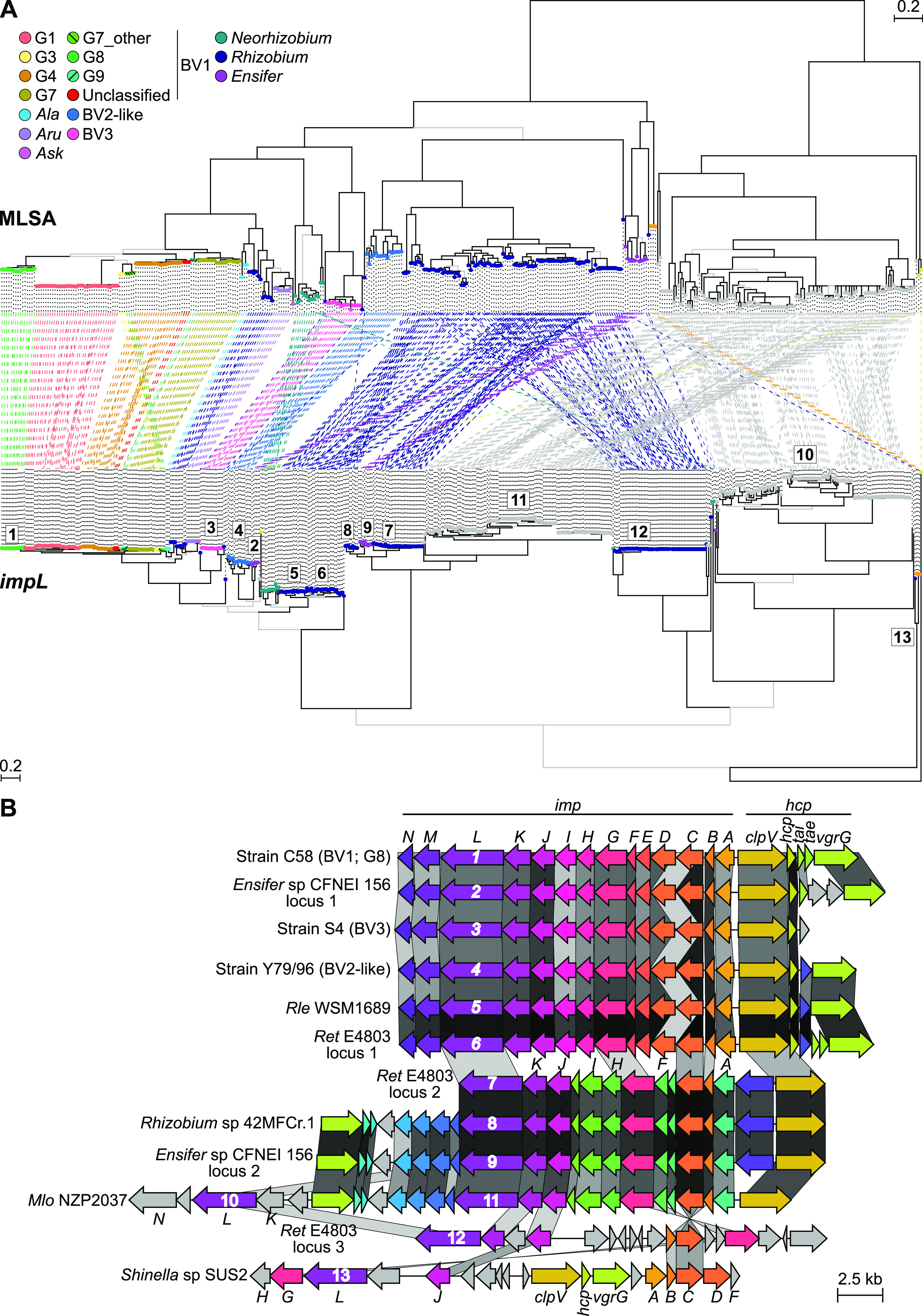
Evolutionary history and structures of T6SS loci. (A) Tanglegram showing incongruencies between strains (top) and *impL* (bottom), a marker gene of the *imp* operon. The MLSA-based tree represents a subset of strains shown in Fig. S1 in the supplemental material. Branches colored in black have ≥70% bootstrap support. For the *impL* tree, branches colored in black exceed 95% UFBoot and 80% SH-aLRT support. Tip points of trees are colored according to the species or lineage classification of the strain. Both trees are midpoint rooted. (B) Synteny plot of *imp* and *hcp* operons. Arrows represent coding sequences and are colored according to gene annotations. Blocks connect homologous genes (white, 0% homology; black, identity). Individual *impL* genes are numbered, and their position is labeled in panel A. Three-letter codes used are Rle, Rhizobium leguminosarum; Ret, Rhizobium etli; and Mlo, Mesorhizobium loti.

We used marker genes conserved in the large T6SS locus to mine for homologous sequences from a large genomic data set previously compiled (19). The locus consisting of the *imp* and *hcp* operons is present in many strains of three major clades of the ARC (Fig. 1; Data Set S1 in the supplemental material). Among agrobacteria, it is present in 91 of 129 genome sequences examined, and evidence suggests the large T6SS locus is inherited mostly vertically, with exceptions in species-level groups G3 and G7_other, a recently identified group in BV1. Evidence reported elsewhere also suggested that in an ancestor of the G1 species-level group, the operons were acquired from an ancestral G8 strain and are located at the end of the linear secondary chromosome, a unique location relative to that of T6SS loci in other BV1-associated species-level groups (22). The large T6SS locus appears to have been lost from an ancestral strain of BV2 and those of some species-level groups within BV1 (Fig. S1). The G2 group is of note because while a large T6SS locus was not identified in genome sequences of 14 G2 strains, the *hcp* operon was identified in a single G2 strain. In LMB-1, the *hcp* locus is present on a contig that includes 180 kb of upstream sequence, but no *imp* operon could be identified on the same or any other contig in its draft genome assembly (34). The large T6SS locus also appears to have been lost more recently by some individual agrobacterial strains, as it has presence/absence polymorphisms in BV2-like and species-level groups G1, G7, and G8 in BV1. Evidence is consistent with recurrent loss at different times in the history of agrobacteria.

10.1128/mBio.01927-21.1 DATA SET S1 Strains analyzed. Download Data Set S1, XLSX file, 0.05 MB. Copyright © 2021 Wu et al. 2021 Wu et al. https://creativecommons.org/licenses/by/4.0/ This content is distributed under the terms of the Creative Commons Attribution 4.0 International license.

10.1128/mBio.01927-21.4 FIG S1 T6SS loci are polymorphic in strains within the ARC. A multilocus sequence analysis-based maximum-likelihood phylogeny of strains of the ARC. The four clades of the genus, as well as major lineages of agrobacteria (bold) and rhizobia, are labeled. Narrow-host-range agrobacteria are abbreviated as Ala, A. larrymoorei,; Ask, A. skierniewicense; and Aru, A. rubi. Red dot indicates the presence of a large T6SS locus. *Mesorhizobium*, *Ochrobactrum*, and Brucella of different families are used as outgroups. The tree is midpoint rooted. Branches colored in black have ≥70% bootstrap support. Download FIG S1, EPS file, 0.6 MB. Copyright © 2021 Wu et al. 2021 Wu et al. https://creativecommons.org/licenses/by/4.0/ This content is distributed under the terms of the Creative Commons Attribution 4.0 International license.

Most members of the clade II rhizobia group (Rhizobium etli, Rhizobium leguminosarum, and Rhizobium phaseoli) have at least one T6SS locus (Fig. 1; Fig. S1; Data Set S1). These are homologous and colinear to either the locus of agrobacteria or one common to strains of *Mesorhizobium*, a genus in a family different than that of the ARC (Fig. 1B). Members of *Ensifer*/*Sinorhizobium* also have homologs of these two large T6SS loci, and evidence is consistent with a more recent gain, as either are present in a very small minority of strains. Last is *Shinella*, in which members have a large T6SS locus that is distantly related and novel in structure relative to others in the ARC. Findings indicate that several variants of the large T6SS locus are present, and they have been relocated, potentially displaced, as well as gained and lost at various times in the history of the genus-level group.

### Agrobacterial strains exhibit different patterns of T6SS regulation.

**FIG 2:**
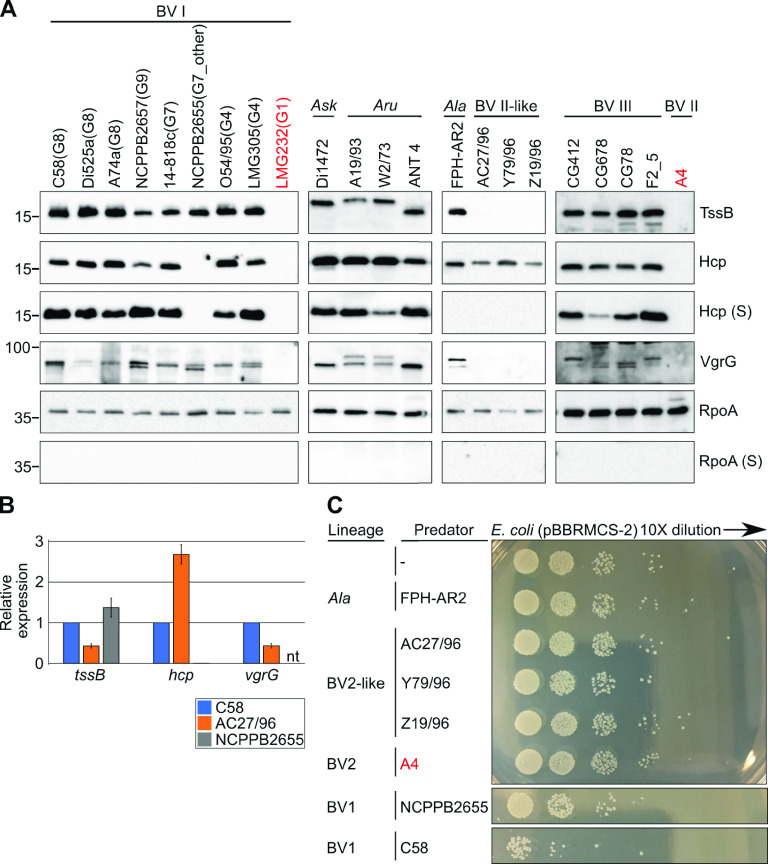
T6SS regulation varies across agrobacteria. (A) Western blotting of T6SS proteins in cellular and supernatant (S) fractions of agrobacterial strains incubated in a minimal acidic medium. For BV1, species-level designations are listed in parentheses after strain names. Three-letter codes used for recognized narrow-host-range species-level groups are Ask, Agrobacterium skierniewicense; Aru, Agrobacterium rubi; and Ala, Agrobacterium larrymoorei. Strains LMG2321 and A4 (labels in red) lack identifiable T6SS-associated loci. The anti-VgrG antibodies can detect both VgrG variants of strain C58. RpoA is a cytoplasmic protein used as a control for cell lysis. Molecular-weight markers (in kilodaltons) are indicated on the left. This is a composite image derived from several western blots. (B) Relative change in expression of T6SS-associated marker genes for strains C58, AC27/96, and NCPPB2655. Cells were grown in an acidic minimal medium, and gene expression was normalized to expression of the 16S rRNA genes and the corresponding gene in strain C58. (C) *In vitro* competition between strains of agrobacteria and E. coli. Strains with different patterns of T6SS expression were competed in the absence of selection and dilution plated to select for E. coli cells. This is a composite image representative of multiple competition assays. All experiments were repeated with similar results.

In agrobacterial strains examined prior to this study, expression of the T6SS is inducible by an acidic minimal medium hypothesized to mimic a plant environment (13, 35, 36). To test whether this response is generalizable across agrobacteria, we examined protein expression patterns of strains representing species-level groups previously unstudied. In reference strain C58, TssB, Hcp, and VgrG, markers for expression of *imp* and *hcp* operons, were expressed when cells were incubated in acidic minimal medium (Fig. 2). In most other tested strains, the three proteins were also expressed and detectable in cellular fractions (Fig. 2) (13). Importantly, Hcp was also detected in their supernatant fractions. When probed with anti-VgrG antibodies, multiple bands were detected in cellular fractions of strain C58 and several others, consistent with predictions of multiple homologs of *vgrG* and evidence that they are coordinately expressed with *imp* and *hcp.* Therefore, most tested strains, when incubated in an acidic medium, secreted marker proteins indicating that their T6SS was activated in a condition shown to activate T6SS in reference strain C58 (35).

Conversely, three strains, each representing a different species-level group, exhibited different patterns of T6SS regulation (Fig. 2A) (19). Strain NCPPB2655 of G7_other expressed TssB and VgrG, but not Hcp, when incubated in acidic minimal medium. Quantitative PCR confirmed that *hcp* was not induced in cells incubated in this medium (Fig. 2B). In contrast, Hcp was detected when cells were incubated in a nutrient-rich medium, demonstrating the *hcp* operon of strain NCPPB2655 is functional (Fig. S2A). Moreover, when its Hcp variant was ectopically expressed in Escherichia coli, it was detectable at similar levels as Hcp of C58, demonstrating that antibodies recognized both variants with similar levels of specificity (Fig. S2B). Findings suggested that in strain NCPPB2655, *vgrG* is under the control of its own promoter and expressed separately from *hcp*. In Agrobacterium larrymoorei strain FPH-AR2, all three marker proteins were expressed when cells were incubated in minimal acidic medium, but the T6SS was not activated (Fig. 2A). Likewise, in BV2-like strain AC27/96, *hcp* was induced, and its protein was present in cellular fractions, but the T6SS was not activated (Fig. 2A and B). Though *tssB* and *vgrG* genes were expressed, neither of their proteins were detected, but this was due to antibody specificity (Fig. 2B; Fig. S2C to D). Strains representing these three species-level groups failed to clearly suppress the growth of E. coli when competed in culture (Fig. 2C). Findings suggest that the T6SS is regulated differently across strains of agrobacteria.

10.1128/mBio.01927-21.5 FIG S2 Specificity of antibodies against different variants of T6SS proteins. Western blot analysis of Hcp in cellular fractions of agrobacterial strains grown in a neutral pH 523 medium (A) and cellular fractions of E. coli heterologously expressing homologs of *hcp* (B). Western blotting of TssB variants expressed in E. coli BL21 (C) and VgrG variants expressed in agrobacterial strain LMG232 (D). In panels A and D, RpoA was used as a loading control. Molecular-weight sizes (left of each panel) are in kilodaltons. All experiments were repeated with similar results. Download FIG S2, EPS file, 0.9 MB. Copyright © 2021 Wu et al. 2021 Wu et al. https://creativecommons.org/licenses/by/4.0/ This content is distributed under the terms of the Creative Commons Attribution 4.0 International license.

### Agrobacteria have diverse T6SS effector genes.

We used *vgrG*, the spike protein-encoding gene, as a query to identify candidate EI pairs because the former is conserved in sequence and its homologs cluster with the latter in genome sequences of agrobacteria. Here, we focused on a subset of genome sequences that correspond to key reference agrobacterial strains and those readily available in our collection. In addition to *vgrG* homologs linked to *hcp*, we identified numerous others in accessory loci (Data Set S2). BV1 strains have from zero to three additional accessory *vgrG* loci, all of which are located on a secondary chromosome. Of those examined, BV2-like strains with a T6SS locus have no accessory *vgrG* loci, and *vgrG* homologs are consistently located adjacent to *hcp* operons on megaplasmids. BV3 strains can have numerous additional accessory *vgrG* loci that can be present on various replicons of their genomes. In the finished genome sequence of strain S4 of BV3, two accessory loci are present on the pAvS4e plasmid, while four are present on the primary chromosome, a pattern observed in no other agrobacterial lineages (23). Like the diversity observed for the large T6SS locus, *vgrG* loci are also highly variable and differ in number, composition, and genomic locations across the three main lineages of agrobacteria.

10.1128/mBio.01927-21.2 DATA SET S2 Predicted *vgrG* loci of agrobacteria. Download Data Set S2, XLSX file, 0.10 MB. Copyright © 2021 Wu et al. 2021 Wu et al. https://creativecommons.org/licenses/by/4.0/ This content is distributed under the terms of the Creative Commons Attribution 4.0 International license.

The 135 identified *vgrG* loci were grouped into one of three sets based on their associated *tap* adapter gene. There are 54 *tap1* (DUF4123)-associated, 66 *tap2* (DUF2169)-associated, and 15 adapter-less *vgrG* loci (Data Set S2). In each *tap1*-associated *vgrG* locus, an EI pair is predicted typically immediately downstream of the adapter gene. All *tap2-*associated *vgrG* loci are associated with a gene encoding a DUF4150 (PAAR-like) domain. In 44 of these, the DUF4150 domain is N-terminal to a polymorphic C-terminal effector domain encoded by so-called specialized effector genes, which encode effectors covalently fused to a component of the T6SS (37). In 22 others, DUF4150 is encoded separately, along with a rearrangement hot spot (RHS) protein, which belongs to a family of large multidomain proteins with polymorphic C-terminal toxin domains known to be associated with T6SS (38). No homologs of DUF1715-encoding adapter genes were identified within a *vgrG* locus of any agrobacterial genome sequence analyzed.

**FIG 3:**
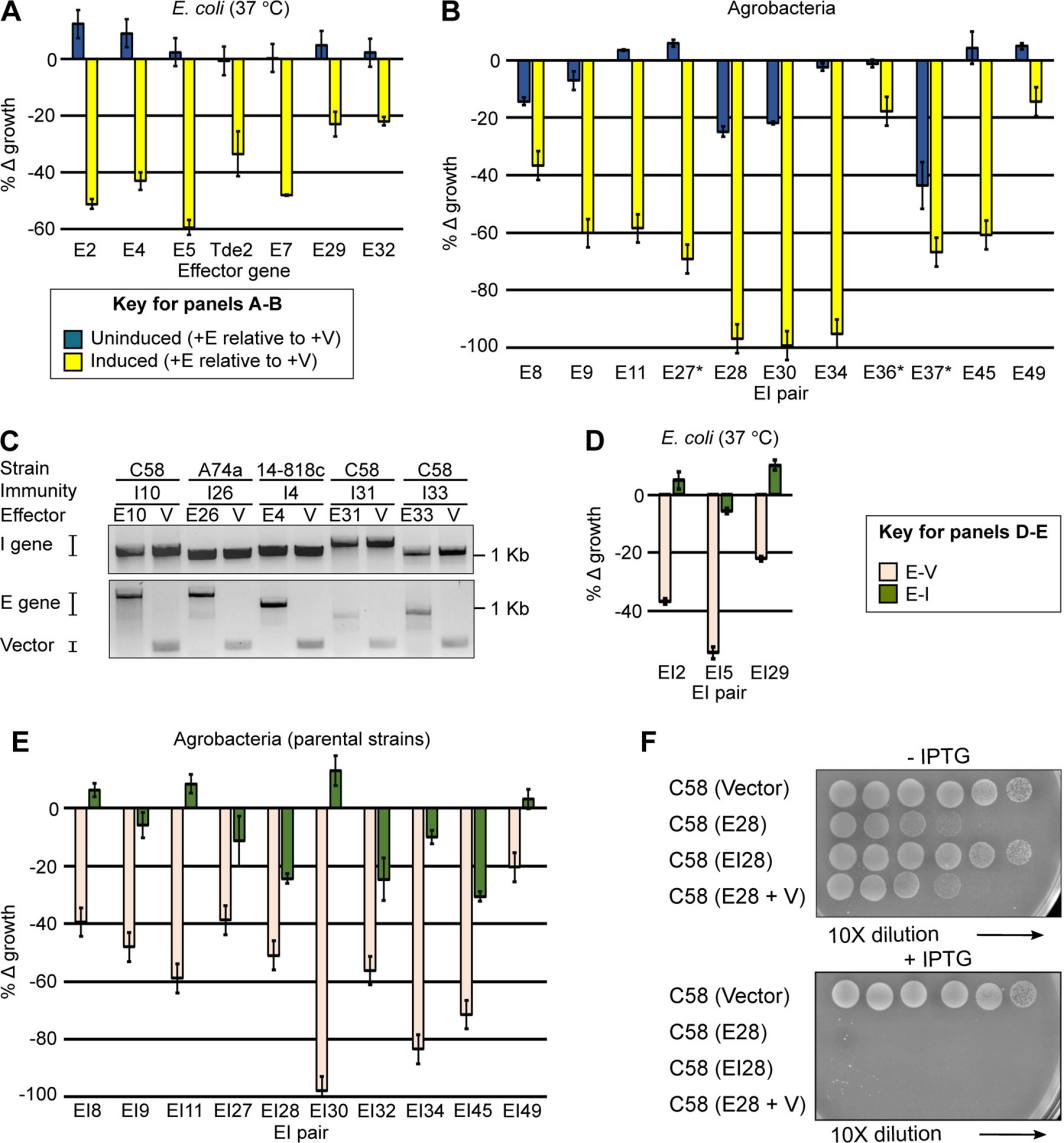
Diverse EI pairs of agrobacteria exhibit toxicity and immunity. Induced expression of effector genes (*x* axis) in E. coli (A) or agrobacteria strain C58 (B) or the parental strain (indicated with an asterisk) of the effector gene caused decreased growth relative (*y* axis) to strains carrying an empty vector (+V) and grown under the same conditions. (C) Some effector genes could not be transformed into agrobacteria unless paired with plasmids carrying cognate immunity genes expressed from constitutive promoters. Inverted image of a 1× Tris-acetate-EDTA (TAE) agarose gel showing surviving colonies were positive for genes transformed into cells. Presence of an immunity gene protected E. coli (D) or agrobacteria (E) from cognate effector genes. Relative changes in growth were calculated as the percent growth in comparison to corresponding strains harboring an effector gene paired with an empty vector. (F) Immunity gene I28 protected C58 against its cognate effector gene when cells were grown on a solid medium in the absence of IPTG. Photographs of colonies 2 days after serially diluted C58 strains were spotting on solid media without or with 1.0 mM IPTG. All experiments were repeated at least two more times and yielded similar results. For growth assays, *n* = 3.

Newly identified candidate effector genes were clustered into 35 families (Data Set S2). A total of 29 candidate effector genes, present within strains in our collection, were selected and tested for toxicity. To our surprise, genes had to be expressed in multiple strains of bacteria, from different promoter/plasmid combinations, and in different conditions to exhibit toxic effects. Some exhibited toxicity to E. coli, strain C58, or the parental strain of the effector gene and/or could not be transformed into bacteria without a cognate immunity gene (Fig. 3A to C). Also, despite being constitutively expressed directly in cells, effectors varied widely in toxicity. When including effector genes previously validated, 26 genes representative of 24 effector families exhibited toxicity in at least one assay (13). In addition, toxicity of 21 effectors was ameliorated completely or partially when their genes were paired with their predicted cognate immunity gene (Fig. 3C to F) (13). Like the toxicity assay, efficacy of immunity genes in conferring protection to cells had to be tested in multiple assays. Collectively, the agrobacterial members of the ARC have many EI pairs that could be reshuffled to generate an enormous number of possible combinations.

Scans of sequences representative of each validated effector family revealed little about their functions. Most effectors were predicted to encode hypothetical proteins. Only five families were identified as potential nucleases, a commonly predicted function for T6SS-associated effectors, while E30 is predicted to be a member of the lysozyme-like superfamily (Data Set S2). Among RHS effectors, those in agrobacteria have one of five different toxic C-terminal domains. Four have domains related to those of Pertussis toxin subunit 1, tricorn protease, thermostable phytase 3-phytase, and calcium-dependent phosphotriesterase (Data Set S2).

We also projected presence/absence patterns of EI pairs, validated and predicted, onto a phylogeny of agrobacteria (Fig. S3). Some EI families are enriched in specific species-level groups or lineages of agrobacteria. For example, EI5 is in nearly 80% of the G1 strains. It is also the case that adapter-less *vgrG* loci, and thus their EI pairs, are exclusive to strains of BV3. Conversely, no EI collection is entirely conserved in any of the well-represented (2+ members) species-level groups or lineages. These two patterns suggest that different species or lineages of agrobacteria have horizontally acquired or evolved unique EI pairs and that interactions among diverse members of agrobacteria further diversify collections through assimilation or displacement of EI pairs.

10.1128/mBio.01927-21.6 FIG S3 Distribution of EI homologs in agrobacteria. A subset of the MLSA-based maximum-likelihood phylogenetic tree is shown on the left (Fig. S1). The tree is midpoint rooted. Branches colored in black have ≥70% bootstrap support. The heatmap shows the presence (black box) or absence (white box) of a homolog of the EI family (experimentally validated or predicted). Download FIG S3, EPS file, 0.6 MB. Copyright © 2021 Wu et al. 2021 Wu et al. https://creativecommons.org/licenses/by/4.0/ This content is distributed under the terms of the Creative Commons Attribution 4.0 International license.

### Recombination occurs at hot spots within *vgrG* loci.

**FIG 4:**
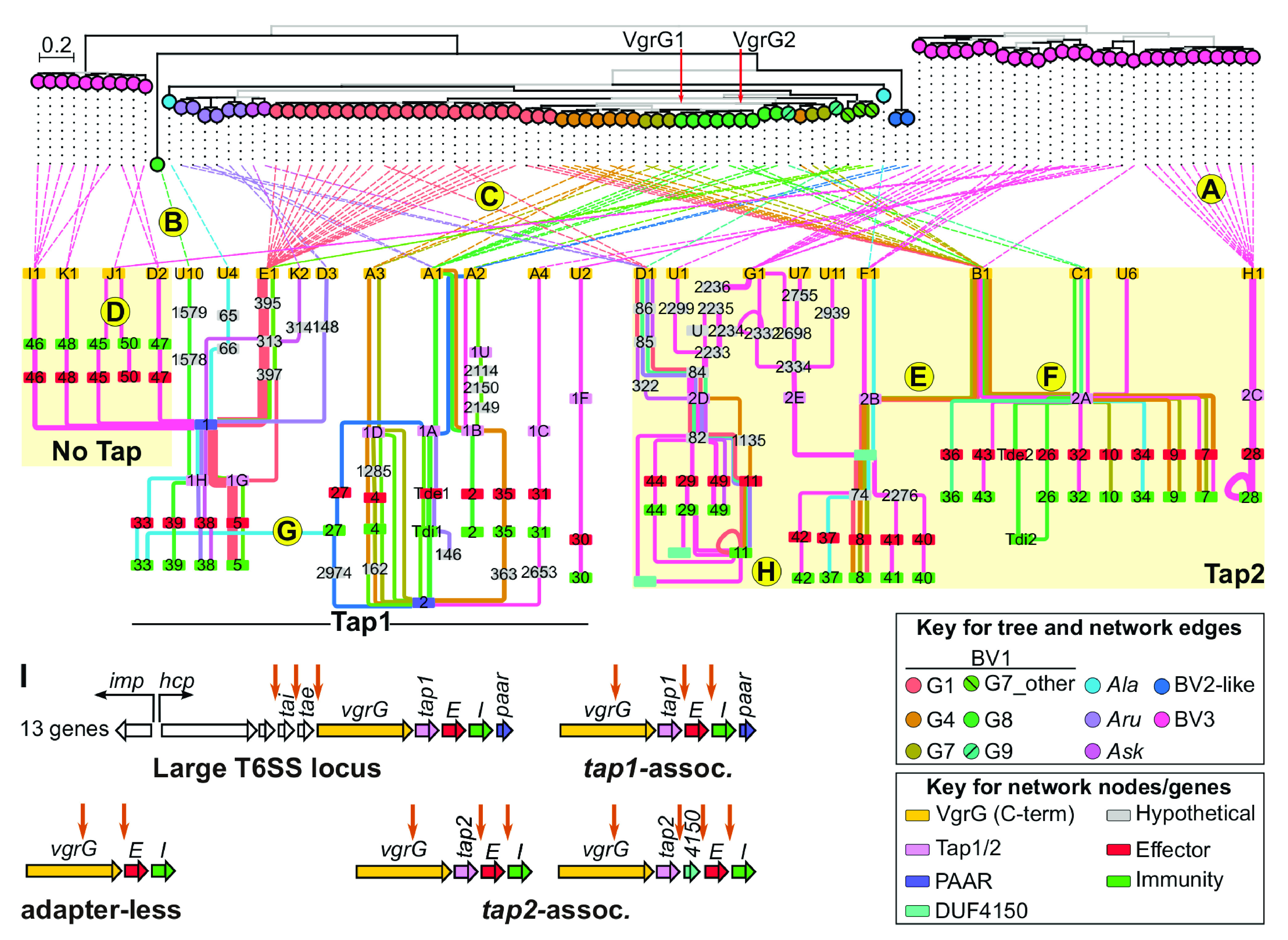
Diversification of *vgrG* loci occurs through multiple processes. A maximum-likelihood phylogenetic tree (top) of the N termini (region upstream of a predicted recombination hot spot) of VgrG variants. The tree is midpoint rooted. Branches colored in black exceed 95% UFBoot and 80% SH-aLRT support. Tip points of the tree are colored according to the species or lineage classification of the strain and linked to their corresponding node in the gene synteny graph of *vgrG* loci (bottom). The *vgrG1* and *vgrG2* homologs of strain C58 are indicated for reference. Patterns indicate vertical inheritance of an entire *vgrG* locus (A), horizontal acquisition of an entire *vgrG* locus (B), recombination within *vgrG* and inheritance of downstream genes (C), recombination downstream of *vgrG* (D and E), recombination downstream of a *tap* gene (F), recombination to yield an orphan immunity gene (G), and recombination or duplication to yield a cluster of immunity gene homologs (H). (I) Diagrams of canonical T6SS loci. (C to H) Downward pointing arrows indicate regions where recombination is inferred to have occurred. Nodes are colored according to predicted function and numbered according to subtype or EI family. The letter “U” indicates a node of a subclassified gene. The DUF4150-encoding genes formed three subtypes that were not numbered. Edges are colored and weighted according to species or lineage and number of members (thinnest line = 1), respectively, with the *vgrG* locus.

To investigate the evolution of *vgrG* loci more deeply, we used polymorphic sequence domains to subclassify *vgrG* and *tap* genes and visualized locus structures in a gene synteny network. VgrG is a modular protein with a conserved COG3501 (TIGR03361) domain in the N-terminal coding portion (residues 17 to 537 of VgrG1 of strain C58), followed by a polymorphic C-terminal domain that confers specificity to effectors (14, 22). We predicted a recombination hot spot within COG3501 and identified up to 18 variable sequence domains in C-terminal coding regions, which were used to subclassify *vgrG* into subtypes (Fig. S4). The *tap* genes also have variable sequence domains, though their relationships to EI families and *vgrG* subtypes are unknown. We constructed a phylogenic tree based on the conserved region of the COG3501 domain and linked tips to nodes corresponding to their *vgrG* subtype in a gene synteny network derived from completely assembled *vgrG* loci with homologs of experimentally validated EI pairs (Fig. 4; Fig. S4). In the tree of conserved regions of COG3501, species-level groups and lineages formed mostly monophyletic clades, suggesting the gene region is largely inherited vertically. As described earlier, *vgrG* loci separate into three sets based on the associated *tap* gene. The network shows that each *vgrG* set is associated with a unique group of EI families (Fig. 4).

10.1128/mBio.01927-21.7 FIG S4 Structural variation of VgrG. (A) *k-*mer graph of full-length *vgrG* homologs. (B) Maximum-likelihood phylogeny of regions upstream of the predicted recombination hotspot of *vgrG.* The tree is midpoint rooted. Branches colored in black exceed 95% UFBoot and 80% SH-aLRT support. Tip points of the tree are colored according to the species or lineage classification of the corresponding strain. (C) Diagrams of predicted sequence domains in translated sequences of *vgrG* and downstream of the predicted recombination hotspot. Homologous domains are colored similarly; colors were randomly assigned to each domain. Download FIG S4, EPS file, 1.4 MB. Copyright © 2021 Wu et al. 2021 Wu et al. https://creativecommons.org/licenses/by/4.0/ This content is distributed under the terms of the Creative Commons Attribution 4.0 International license.

Despite the overarching structure, there are multiple patterns reflecting processes that have shaped the evolution of *vgrG* loci. As a reference point, we first highlight the simplest case of vertical inheritance of an entire *vgrG* locus (Fig. 4A). In this example, there is a monophyletic clade of *vgrG-H1* linked to a single subnet in the network. The subnet shows that in this data set, the locus is exclusive to BV3 and organized with *vgrG-H1* followed by *tap2C* and EI28, in which I28 is duplicated. Horizontal acquisition of an entire *vgrG* locus is reflected as a COG3501 sequence that neither clusters with its most closely related homologs nor links to the same subnet of other species members (Fig. 4B). We observed fewer instances of this than originally predicted, but inspection of *hcp* operons suggested that 3′ regions are variable in composition and have potential recombination hot spots to swap entire *vgrG* loci (Fig. 4I; Fig. S5) (13). The potential for a complete *vgrG* locus to displace the 3′ region of the *hcp* operon is consistent with expression patterns uncovered in strain NCPPB2655 (Fig. 2).

10.1128/mBio.01927-21.8 FIG S5 Recombination in the *hcp* operon. Multiphylogeny of marker genes for the *imp* and *hcp* operons. Lines connect nodes of physically linked genes. Downward-facing red arrows indicate patterns consistent with recombination. Tip points of trees and connecting lines are colored according to the species or lineage classification of the corresponding strain. All trees are midpoint rooted. Branches colored in black exceed 95% UFBoot and 80% SH-aLRT support. Download FIG S5, EPS file, 1.0 MB. Copyright © 2021 Wu et al. 2021 Wu et al. https://creativecommons.org/licenses/by/4.0/ This content is distributed under the terms of the Creative Commons Attribution 4.0 International license.

Recombination is also predicted to occur within *vgrG* loci. The most prominent is one within *vgrG* itself that is reflected as members of a monophyletic clade of COG3501-encoding domains linking to diverse subnets (Fig. 4C and I). Such a pattern is indicative of a fragment, encompassing the C-terminal coding portion of *vgrG* and all genes downstream of the locus, recombining and displacing the original locus. There are also patterns in which *vgrG* subtypes and the *tap* gene have edges to multiple nodes (Fig. 4D and F to I). For example, the adapter-less *vgrG-J1* subtype is linked directly to EI45 and EI50, and *tap2-A* is linked to nine different families of EI pairs (Fig. 4D, F, and I). We suggest this reflects recombination involving regions distal to *vgrG* in *vgrG* loci. Last, recombination of even smaller fragments, or possibly duplication, can yield orphan immunity genes and clusters of immunity gene homologs (Fig. 4G to I). As previously demonstrated, only one of the three homologs of I11 protects cells against E11, and we thus cannot distinguish between duplication followed by diversification or acquisition of a homolog from a *vgrG* locus with a different yet homologous effector gene (13).

HGT and recombination have potential to greatly diversify *vgrG* loci. Indeed, entire *vgrG* loci have been shuffled broadly across the genus. Likewise, recombination within COG3501 has also shuffled loci broadly and transitioned loci from one adapter type to another. In strain C58, evidence supports a scenario where a *tap1-* or *tap2-*associated locus was converted to the other, as *vgrG1* and *vgrG2* have closely related COG3501-encoding domains. Likewise, some members of the *vgrG-J1* subtype of BV3 have COG3501 domains that cluster with those of *vgrG* homologs that link to *tap1.* However, when recombination is inferred to occur downstream of *vgrG*, all edges in the network are restricted to within one of three main sets of *vgrG* loci, with no edge crossing between sets (Fig. 4) (14). This pattern indicates that selection acts to preserve genetic links between specific subtypes of *vgrG*, *tap*, and EI families. Consequently, the location where recombination occurs can limit which recombining loci will yield a functional *vgrG* locus.

**FIG 5:**
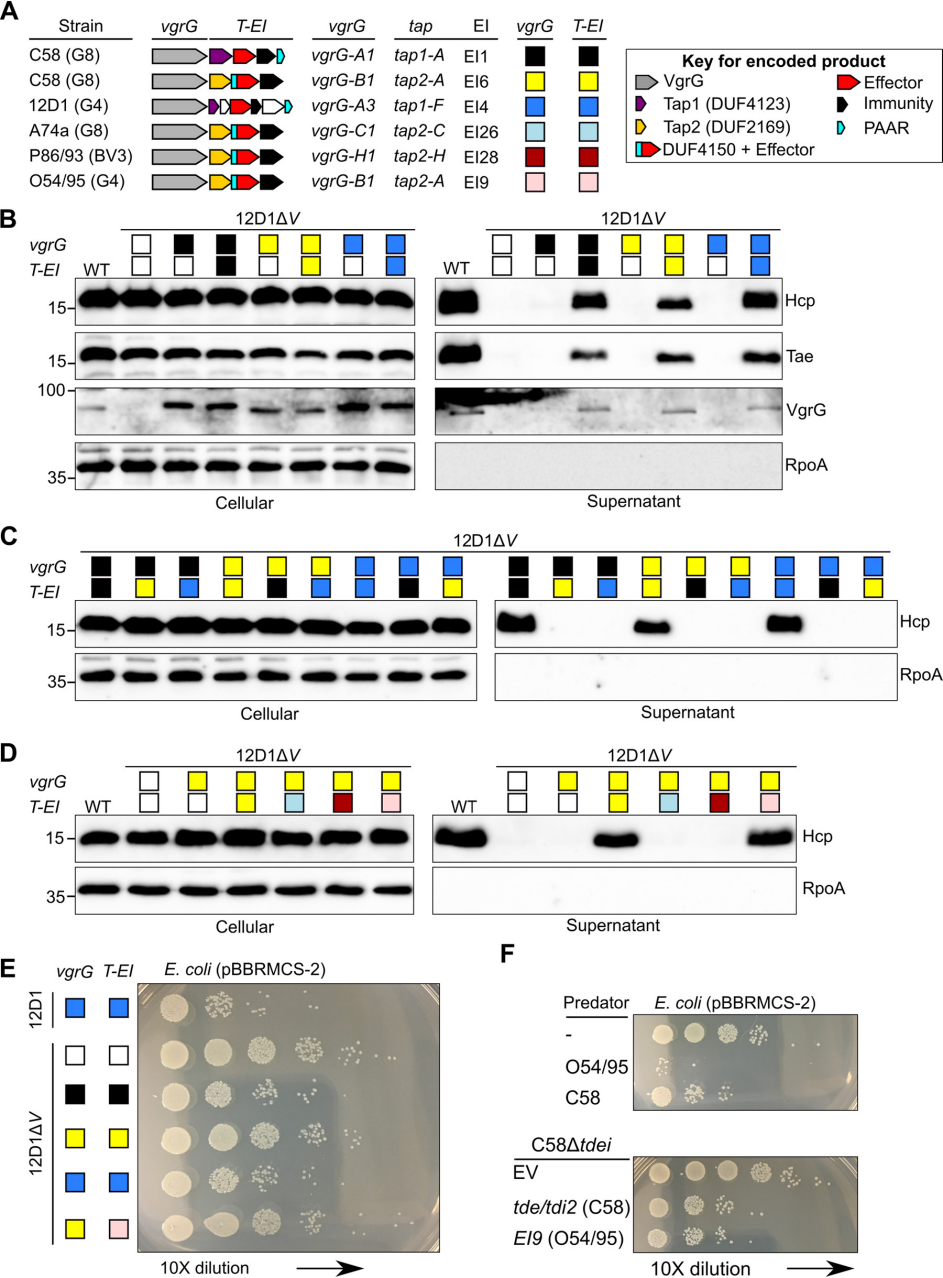
Gene subtype combinations influence T6SS function. (A) Schematic of *vgrG* loci and colors used to symbolize plasmids with *vgrG* as well as *tap* and EI gene pair (*T-EI*). (B) Western blot analysis of cellular and supernatant fractions of 12D1Δ*V* harboring *vgrG* alone or paired with its native combination of *tap* and EI pair. (C and D) *vgrG* paired with its native or nonnative combination of *tap* and EI pair. RpoA is a cytoplasmic protein used as a control for cell lysis. Molecular-weight markers are indicated in kilodaltons. All western blot analyses were repeated twice with similar results. (E and F) Interbacterial competition assays between agrobacterial strains (predator) and E. coli (prey), mixed at a ratio of 30:1 (predator to prey). Cells were serially diluted and spotted onto LB agar selective for E. coli and grown overnight prior to photographing. The assays were repeated at least three times in two independent experiments and yielded similar results.

To experimentally test the extent to which the composition of sequence domains in genes influences locus diversification, we first deleted the only *vgrG* locus from strain 12D1 to generate 12D1Δ*V*, a disarmed strain that fails to secrete Hcp and antagonize E. coli (Fig. 5A, B, and E) (13, 39). We then built one set of constructs consisting of various *vgrG* subtypes and another set of constructs consisting of *tap* and cognate EI pairs (*T-EI*; Fig. 5A). Reconstitution of combinations native to strains 12D1 and C58 restored the capacity for 12D1Δ*V* to activate its T6SS and secrete proteins (Fig. 5B). However, no nonnative combination from the three *vgrG* loci of the two strains restored T6SS activity in 12D1Δ*V* (Fig. 5C). Most notable was the failure of two combinations derived from within *tap1*-associated *vgrG* loci to complement 12D1Δ*V*. Likewise, two nonnative combinations derived from within *tap2*-associated *vgrG* loci failed to complement 12D1Δ*V* (Fig. 5D). In contrast, one nonnative combination that preserved the link between *vgrG-B1* and *tap2-A* gene subtypes successfully complemented 12D1Δ*V* for secretion of Hcp (Fig. 5D).

The capacity for strains with reconstituted *vgrG* loci to compete with bacteria was tested. However, the 12D1Δ*V* strain carrying gene combinations that reconstituted a T6SS functional in secretion exhibited weak antagonism against E. coli in culture (Fig. 5E). This was not surprising, as wild-type strain 12D1 has weak T6SS-dependent activity in culture and is incapable of competing in a T6SS-dependent manner *in planta* against other agrobacterial strains (13). Therefore, as an alternative test, we transformed *EI9* cloned from a *vgrG-B1-tap2A* locus of strain O54/94 into strain C58*tdei.* This mutant strain retains *vgrG-A1-tap1-A* and *vgrG-B1-tap2A* loci but lacks both of their associated EI pairs. As predicted, *EI9* complemented the C58*tdei* and suppressed growth of E. coli by approximately 1,000× (Fig. 5F). Findings demonstrate that specificity at the level of subtypes among *vgrG*, *tap*, and effector-encoding genes is necessary to ensure proper functioning of the T6SS.

## DISCUSSION

Here, we revealed the diversity of T6SSs among members of a genus of Gram-negative bacteria. Like other genera and some species, the agrobacteria-rhizobia complex has multiple variants of *imp* and *hcp* that have been gained and lost recurrently. For example, eight different T6SSs have been identified from *Burkholderia*, with strains encoding up to six different variants, while three T6SS with different evolutionary histories are present in Pseudomonas aeruginosa strains (40–42). In these two taxa, T6SS can have specific roles in virulence toward eukaryotes or in interbacterial competition. In agrobacteria, the large T6SS loci of members are inferred to have a common evolutionary history (Fig. 1). Hence, it was unexpected to discover that not all strains deploy their T6SS under the same condition (Fig. 2). This raises the possibility that interbacterial competition is triggered by different signals and is important during different stages of the agrobacterial life cycle. It is also conceivable that some strains or species of agrobacteria use a T6SS to acquire nutrients, as not all *vgrG* loci were confirmed to include an EI pair (43, 44). Whether agrobacteria deploy T6SS against eukaryotes remains unknown. Across the diverse lineages of agrobacteria, we identified and experimentally confirmed at least 24 effector families and showed that effector genes are organized downstream of *vgrG* and linked to one of two adapter-encoding genes or no identifiable adapter-encoding gene (Fig. 3). The organization of *vgrG* loci is important for promoting diversification, but variation in composition limits the number of recombinants that will yield functional gene combinations and thus constrains diversification.

Horizontal gene transfer is fundamental to the evolution of bacteria and is implicated in diversifying EI collections. HGT of effectors is often inferred based on the patchy distribution of their genes or linkage of genes to mobile genetic elements (8, 45). Computational and experimental studies have demonstrated that in Vibrio cholerae, entire accessory *vgrG* loci can be acquired, sometimes displacing the original locus (7, 10). Findings here indicate that acquisition of entire *vgrG* loci has occurred in agrobacteria (Fig. S5 in the supplemental material). In addition, duplication, followed by recombination, is another potential mechanism for diversifying EI collections in agrobacteria (33). Some *vgrG* loci of agrobacteria are associated with plasmids and are potentially acquired by conjugation. Likewise, in Myxococcus xanthus, effector genes are located within prophage-like elements and are thus easily mobilized (46). Agrobacteria are also suggested to be naturally competent, which, when coupled with T6SS-mediated competition, provides cells the ability to promote the availability and uptake of environmental DNA (7, 47, 48). Conjugation and natural competence are both implicated in the acquisition of large amounts of DNA, as megaplasmids can be exchanged through conjugation, and in V. cholerae, T6SS-mediated antagonism is associated with the acquisition of DNA averaging 50 kb in size (49). Therefore, transmission of entire large T6SS loci and accessory *vgrG* loci is possible. In the ARC, distribution patterns of conjugative plasmids are consistent with distantly related lineages interacting and exchanging DNA (25).

Modularity is an important property of T6SSs, as it allows components to organize at multiple levels and evolve semiautonomously, preserving functionality while conferring flexibility (50). For example, RHS and specialized effectors, also in agrobacteria, allow for reshuffling of toxin domains downstream of a conserved sequence while maintaining functions necessary for T6SS assembly and activation (51, 52). In addition, genes necessary for effector loading, a final checkpoint that regulates T6SS assembly and activation, are consistently clustered as a functional unit in *vgrG* loci (39, 53). This facilitates seamless assimilation of an entire unit with the secretion machinery encoded by recipient bacteria. Modularity also enables acquired loci to recombine and generate new gene combinations. Recombination is suggested to occur anywhere within *vgrG* loci of V. cholerae (17). In *Pantoea* and *Erwinia*, analysis of changes in GC%, a signature of HGT, supported the possibility of recombination within *vgrG* (54). Here, we coupled a phylogenetic tree to a network to visualize effects of recombination in *vgrG* and relationships between gene subtypes of agrobacteria to inform on features that not only promote EI diversification but also limit it (Fig. 4). Selection acting to preserve links between subtypes of genes encoding specific domains counteracts flexibility (Fig. 5; Fig. S4). Consequently, recombination within loci likely occurs mostly in hot spots, and, as the distance between hot spot location and *vgrG* increases, there is an increasing chance to disrupt T6SS functionality. Thus, recombination distal to *vgrG* is more likely to preserve a functional combination if between loci that are similar in composition and organization. Furthermore, *vgrG* loci of agrobacteria often have additional and polymorphic genes encoding predicted hypothetical proteins. Such genes are present in loci of other bacterial taxa, and in the few cases that have been tested, evidence suggests that they are required to ensure the proper functioning of effectors (55, 56). Therefore, it is likely that as the complexity of *vgrG* loci increases, flexibility decreases.

Agrobacteria are important bacteria because of their capacity to genetically transform plants and cause disease or be disarmed and used to modify plants. Characterization of reference strain C58 has generated a foundation of knowledge on possible ecological roles of the T6SS for plant-associated bacteria (28). As revealed here and elsewhere, members of the genus vary in the presence and regulation of T6SS as well as in their strength of interbacterial competition (13, 22, 33). T6SSs have diverse roles in the genus, underscoring the importance of uncovering and understanding the natural variation in traits to improve plant health and uses of agrobacteria in plant research and biotechnology.

## MATERIALS AND METHODS

### Bioinformatics and genome analyses.

Genome sequences used for most analyses were described previously (Data Set S1 in the supplemental material) (19). The multilocus sequence analysis-based phylogenetic tree of *Rhizobiaceae* was also previously described (19).

GET_HOMOLOGUES v3.12 with the options “-M -t 0” was used to cluster translated sequences of genes into orthologous groups (57). The HMMER v3.3 program hmmsearch and the Pfam or TIGRFAM hidden Markov models (HMMs) of VgrG (TIGR03361), DUF4123 (PF13503.2), DUF2169 (PF09937.5), DUF1715 (PF09811.11), PAAR_motif (PF05488.9), DUF4150 (PF13665.3), GAD-like (PF08887.7), FHA (PF00498.22), Ntox15 (PF15604.2), and RHS/RHS_repeat (PF03527.16; PF05593.16) were used to assign putative functions to ortholog groups (58).

To predict T6SS-associated genes, the sequences of members of ortholog groups defined as VgrG, as well as proteins encoded by genes of the *imp* and *hcp* operons, were used to mine translated genome sequences of ARC members. The presence of the large T6SS locus was identified based on its corresponding strain being included in ortholog groups of T6SS-associated proteins and/or identification of *impC* (Atu4341), *impL* (Atu4332), and *vgrG* (Atu4348) homologs following BLASTp searches of translated genome sequences (59). BioPython was used to extract sequences of large T6SS loci, and clinker v0.0.20 with default options was used to visualize levels of homology and synteny (60, 61).

To predict *vgrG* loci of agrobacteria, we identified *vgrG* and all downstream genes up to a gene on the opposite strand or a homolog of a conserved non-T6SS-associated gene (Atu4353). The annotations and predicted domains of genes downstream of *vgrG* were manually inspected to identify putative adapter- and PAAR-encoding genes. Candidate EI pairs were identified based on their location relative to VgrG and DUF4123- or DUF2169-encoding genes. Some *vgrG* loci were excluded from analyses because of misassemblies due to *vgrG* homologs collapsing into one sequence.

For all effector families with at least one experimentally validated member, InterProScan v5.48-83.0, with the default parameters, was used to annotate translated sequences for all members of each family (62).

To construct and visualize the gene synteny network for agrobacterial *vgrG* loci, we used Cytoscape v3.8.2 and previously described methods (19, 63).

For phylogenetic analysis, MAFFT v7.471 with the default options was used to generate multiple-sequence alignments (64). IQ-TREE v1.6.12 with the options “-bb 1000 -alrt 1000” was used to generate phylogenetic trees (65). The R package ggtree v2.99.0 was used to visualize individual phylogenies and the multitree comparison (66). The R package phytools v0.7-70 function “cophylo” was used to plot tanglegrams of trees (67).

To predict recombination breakpoints in *vgrG*, we used GARD with the default options and an alignment of translated sequences as input (68).

To identify sequence motifs in the translated sequences of *vgrG* and *tap* genes, we used MEME v5.3.3 with the options “-mod zoops -nmotifs 25 -minw 30 -maxw 300 -objfun classic -markov_order 0” (69). The R package gggenes v0.4.1.9002 was used to visualize subdomains of VgrG (70).

The program TwoPaCo v0.9.2 with the options “-f 40 -k21” was used to generate a *k*-mer graph of agrobacterial *vgrG* sequences, which was then visualized using Bandage v0.8.1 (71, 72).

### Bacterial strains and growth conditions.

Strains of A. tumefaciens were grown at 28°C in either LB or 523 media (75). E. coli DH10B was grown at 37°C in LB (Invitrogen, Carlsbad, CA, USA). The antibiotics gentamicin, spectinomycin, and kanamycin were used as required at concentrations of 50 μg/ml, 100 μg/ml, and 30 μg/ml, respectively.

Previously published methods were followed for competition assays in culture (13).

### Plasmid and mutant construction.

Gene coding sequences were PCR amplified and recombined to pDONR222 via Gateway BP reaction (Invitrogen, Carlsbad, CA, USA). Genes from entry clones were recombined via Gateway LR reaction into pJN105_RfC.1, pRL662_RfC.1, pTrc200_RfC.1, or pDEST17 (13) (Invitrogen, Carlsbad, CA, USA). Recombined plasmids were verified via PCR.

To construct the plasmid for generating the 12D1Δ*V* deletion mutant, fragments of approximately 500 nucleotides in length and flanking the targeted genomic region were amplified in PCRs. The Gibson assembly method was used to ligate the two flanking fragments to pJQ200KS, and the final plasmids were transformed into E. coli strain DH10B (NEB, Ipswich, MA, USA). Previously described methods were followed to generate the deletion mutant in strain 12D1 (73).

Sequences of primers used in this study are listed in Data Set S3.

10.1128/mBio.01927-21.3 DATA SET S3 Sequences of oligonucleotides used. Download Data Set S3, XLSX file, 0.02 MB. Copyright © 2021 Wu et al. 2021 Wu et al. https://creativecommons.org/licenses/by/4.0/ This content is distributed under the terms of the Creative Commons Attribution 4.0 International license.

### Reverse transcription-quantitative PCR.

Agrobacterial strains were grown overnight, centrifuged (10,000 × *g*, 10 min), and resuspended at optical density at 600 nm (OD_600_) of 0.2 in AB-MES medium (pH 5.5) (76). After shaking for 4 h at 28°C, cells were harvested. Total RNA was isolated using the RNeasy minikit (Qiagen, Valencia, CA, USA), and residual DNA was removed using DNase I (NEB, Ipswich, MA, USA). Moloney murine leukemia virus (M-MLV) reverse transcriptase (Promega, Fitchburg, WI, USA) and random-hexamer oligonucleotides were used to convert 2 μg of RNA from each sample into cDNA. Quantitative RT-PCRs were done in 10-μl mixtures containing 1 μl 4-fold diluted cDNA, 5 pM each of the forward and reverse gene-specific primers, and 5-μl SsoAdvanced universal SYBR green supermix (Bio-Rad, Hercules, CA, USA) and monitored using a C1000 thermal cycler with CFX96 real-time detection system (Bio-Rad, Hercules, CA, USA). Each sample was done in triplicate. Expression was determined using the threshold cycle (2^−ΔΔ^*^CT^*) method, relative to 16S rRNA of the corresponding strain and the homolog of strain C58 (74).

### Protein expression and Western blotting.

For T6SS expression and secretion analysis, agrobacterial strains were grown overnight, centrifuged (10,000 × *g*, 10 min), and resuspended at OD_600_ of 0.2 in AB-MES medium (pH 5.5) or 523 medium (pH 7.0). For heterologous expression of TssB and Hcp, overnight cultures of E. coli BL21(DE3) were diluted 1:10 in 5 ml LB broth. Cells were induced with 0.5 mM IPTG (isopropyl-β-d-thiogalactopyranoside) for 2 h at 28°C. For heterologous expression of VgrG in strain LMG232, cells containing pTRC200::*vgrG* from strain AC27/96 or strain C58 were grown in AB-MES medium (pH 5.5) for 4 h at 28°C. Bacterial cell lysates were prepared as previously described (73).

After shaking for 4 h at 28°C, cellular and extracellular proteins were separated by centrifugation and prepared as previously described (73). Briefly, 30 μl 1% sodium deoxycholate and 150 μl trichloroacetic acid were added to 1.0 ml of extracellular fraction. The precipitated proteins were resuspended in 20 μl SDS-protein sample loading buffer. The cellular fractions were resuspended in 1 × SDS-protein sample loading buffer at an OD_600_ of 5.0. Total and supernatant fractions were separated on SDS-PAGE gels and transferred to PVDF (polyvinylidene difluoride) membranes.

Western immunoblots were probed with primary polyclonal antibodies recognizing Hcp, RpoA, TssB, Tae, or VgrG proteins (27, 29). Horseradish peroxidase-conjugated goat anti-rabbit IgG secondary antibody was used for detection. The West Pico Plus chemiluminescence substrate (Thermo Fisher Scientific, Waltham, MA, USA) was used to detect Western blots.

### Effector and immunity protein assays.

For heterologous expression of effector and EI pairs, overnight cultures of E. coli DH10B cells containing either pJN105::effector gene or an empty pJN105 vector were resuspended in 100 μl LB broth to an OD_600_ of 0.1 with or without 0.02% l-arabinose and transferred in triplicate to a 96-well microtiter plate. To express effector and EI gene pairs in agrobacterial strains, overnight cultures of cells with either pTrc200::effector gene or an empty pTrc200 vector were resuspended to an OD_600_ of 0.1 with or without 1 mM IPTG in 100 μl of 523 broth and transferred in triplicate to a 96-well plate. A Tecan Spark 10M plate reader was used to measure the OD_600_ of cultures every hour for 10 h (Tecan, Switzerland). E. coli and agrobacterial strains were grown at 37°C/28°C and 28°C, respectively. To calculate relative growth inhibition, the averaged OD_600_ values at 8 and 10 h of culturing for E. coli cells carrying pJN105::effector gene and agrobacterial strains carrying pTrc200::effector gene, respectively, were calculated in comparison to cells carrying empty vectors and grown in the corresponding noninducing or inducing condition.

For immunity assays, the OD_600_ values at 8 h of culturing of E. coli cells were averaged within each treatment, and values of E. coli cells containing pJN105::EI gene pairs were calculated as percentage relative to E. coli carrying pJN105 growing under inducing conditions. For immunity in agrobacterial strains, the OD_600_ at 10 h of cultures were averaged and used. For EI34_FPH-AR2_, E34 or EI34 were cloned into pTrc200 and assayed in C58 cells. For EI28_T268/95_, C58 carrying either pTrc200::effector gene with pRL662::immunity gene or an empty vector were serially diluted and spotted onto LB plates with or without IPTG. For EI4_14-818c_, EI10_14-818c_, EI26_A74a_, EI31_CG412_, and EI34_FPH-AR2_, the pTrc200::effector could not be transformed into C58 or parental strains or could only be cloned in the presence of the cognate immunity constitutively expressed from vector pRL662.

### Data availability.

Network graphs in Cytoscape format and phylogenetic trees in Newick format can be downloaded from https://github.com/osuchanglab/T6SSManuscript.
